# Validation of Skully Care as a Fast Method for Quantifying Positional Cranial Deformities

**DOI:** 10.1177/10556656211035022

**Published:** 2021-09-24

**Authors:** Léon N.A. Van Adrichem, Sophia A.J. Kronig, Otto D.M. Kronig

**Affiliations:** 1 Department of Plastic and Reconstructive Surgery and Hand Surgery, 8124 University Medical Center Utrecht, the Netherlands

**Keywords:** positional skull deformation, shape analysis, computer-assisted diagnosis, validation, asymmetry in infancy, plagiocephalometry

## Abstract

**Objective:**

Craniofacial measuring is valuable for diagnosis and evaluation of growth and treatment of positional skull deformities. Plagiocephalometry (PCM) quantifies skull deformities and is proven to be reliable and valid. However, PCM needs direct skin contact with thermoplastic material, is laborious and time-consuming. Therefore, Skully Care (SC) was developed to measure positional skull deformities with a smartphone application.

**Design:**

SC is retrospectively compared to PCM.

**Setting:**

Pediatric physiotherapy centers.

**Patients:**

Age ≤1 year, analyzed or treated for positional skull deformities.

**Interventions:**

A total of 60 skull shape analyses were performed.

**Main Outcome Measures:**

The main outcome measures employed are Pearson correlation coefficient between cranial vault asymmetry index (CVAI; in SC) and oblique diameter difference index (ODDI; in PCM) and between cranial index (CI; in SC) and cranial proportional index (CPI; in PCM). Mann–Whitney *U* test determined difference of time consumption between PCM and SC.

**Results:**

High correlation was found between CVAI and ODDI (*r *= 0.849; *P *< .01) in positional plagiocephaly and very high correlation between CI and CPI (*r* *=* 0.938; *P *< .01) in positional brachycephaly. SC is significantly faster than PCM (*P *< .001).

**Conclusions:**

SC is valid in analyzing positional skull deformities and strongly correlates to PCM, the gold standard in daily physiotherapy practice. The combination of simplicity, validity, speed, and user and child convenience makes SC a promising craniofacial measuring method in daily practice. SC has potential to be the modern successor for analyzing positional skull deformities.

## Introduction

Positional skull deformities in newborns and young children are a common problem in daily clinical practice (van Vlimmeren et al., 2006). The primary diagnosis of positional skull deformities, such as positional plagiocephaly, positional brachycephaly, and positional scaphocephaly, is based on clinical history taking and physical examination. In the case of suspected premature fusion of cranial sutures (craniosynostosis), the Dutch guideline “treatment and care for craniosynostosis” recommends computed tomography scanning (Mathijssen, 2015). However, in positional skull deformities, physical examination alone is not accurate enough for outcome measurement. Therefore, several methods of quantification were proposed. Firstly, the Argenta classification is used for the establishment of severity of the skull shape; however, this method is a visual assessment and is therefore subjective and not quantitative (Argenta et al., 2004; Feijen et al., 2012). Secondly, caliper measurement is frequently used in the clinical setting; this method is objective, but the interrater reliability of caliper measurements ranges widely between studies (Graham et al., 2005; Mortenson and Steinbok, 2006).

Due to the aforementioned limitations of used methods for the quantification of severity, plagiocephalometry (PCM) was introduced in 2005. In PCM, a thermoplastic strip is positioned around the infant's head at the widest transverse circumference, landmarks are placed on the ring, and following several lines are drawn and measured (van Vlimmeren et al., 2006). Following a reliability and validation study, this method is proven to be both reliable and valid for research and treatment purposes of positional skull deformities (van Vlimmeren et al., 2006, 2008; van Adrichem et al., 2008; van Wijk et al., 2014). However, PCM is laborious and time-consuming.

Hence, Skully Care (SC) was developed as a faster, user- and child-friendly craniofacial measuring method. SC analyzes skull shape by using a standardized photograph from above (vertex view) in an application on a smartphone. This photograph is analyzed digitally and both shape and severity of the deformity are established.

In the current paper, the aim is to validate SC as a craniofacial measuring device by comparing it to PCM. Validation is performed on newborns and young children in physiotherapy practices.

## Methods

SC and PCM measurements are performed in pediatric physiotherapy centers on infants diagnosed with positional skull deformities.

### Patients

For the purposes of this study, we conducted 60 skull shape analyses on children (age ≤ 1 year) with positional skull deformities. The children were analyzed and treated at pediatric physiotherapy centers in Zeist, Tiel, and Woerden (the Netherlands). In the Dutch healthcare system, children are regularly treated at primary child healthcare centers according to a national protocol. If children are suspected for positional skull deformations, they are referred to pediatric physiotherapy centers. It is recommended to measure the degree of deformation by PCM. (Dutch Center Child Healthcare. Nederlands Centrum Jeugdgezondheid [JGZ] guideline, prevention, signalizing, and treating positional preference and skull deformation. In Dutch, JGZ-richtlijn Preventie, signalering en aanpak van voorkeurshouding en schedelvervorming. Utrecht: TNO (the Netherlands Organisation for applied scientific research; in Dutch, Nederlandse organisatie voor Toegepast Natuurwetenschappelijk Onderzoek) innovation for life; 2012; https://www.ncj.nl/richtlijnen/alle-richtlijnen/richtlijn/voorkeurshouding-en-schedelvervorming). To be eligible for inclusion, PCM and SC had to be performed. Analysis was performed in the diagnostic phase and during treatment by 3 experienced and registered pediatric physiotherapists. All measurements were performed in the same visit. Children were referred by primary child healthcare centers and general practitioners. In 10 cases, duration of measurement of PCM and SC was established. Retrospectively we started analyzing those data in September 2020. Based on the validation study of PCM (21 patients), we hypothesized that 60 comparative measurements should be enough to prove a clinical relevant correlation (van Adrichem et al., 2008). We started including patients measured in June 2020 and went back in time till we included 60 patients. It turned out to be a period of 9 months (October 2019 till June 2020).

### Plagiocephalometry

PCM was performed using a strip of thermoplastic material (3.2 mm thick) of dimensions 18 mm × 50 cm heated to 60 °C, which was positioned around the infant's head at the widest transverse circumference and cooled. Three landmarks are placed perpendicular on the strip at the middle of the dorsum of the nose and the posterior edge of the tragus of both ears. A fourth landmark is placed at the middle of the posterior circumferential distance between the ear marks. Based on the landmarks, the 40° oblique diameter difference index (ODDI) as a parameter for positional plagiocephaly and the cranial proportional index (CPI) as a parameter for positional brachycephaly are calculated (van Vlimmeren et al., 2006; van Adrichem et al., 2008). A severity scale is connected to PCM (van Vlimmeren et al., 2008; van Wijk et al., 2014). ODDI as a parameter for positional plagiocephaly is divided in 4 levels of severity: normal < 104; mild ≥ 104 and <108; moderate ≥ 108 and <112; severe ≥ 112. CPI as a parameter for positional brachycephaly is divided in 4 levels of severity: normal <  90; mild ≥ 90 and <95; moderate ≥ 95 and <100; severe  ≥ 100.

### Skully Care

During the same consultation at the outpatient clinic, standardized photographs were taken with the SC application. This SC application (www.skullycare.com) is installed on a smartphone. The SC application is hosted on a certified server (ISO/IEC 27001:2013) and acts according to the law of protection of personal data (General Data Protection Regulation, the Netherlands).

**Figure 1.**
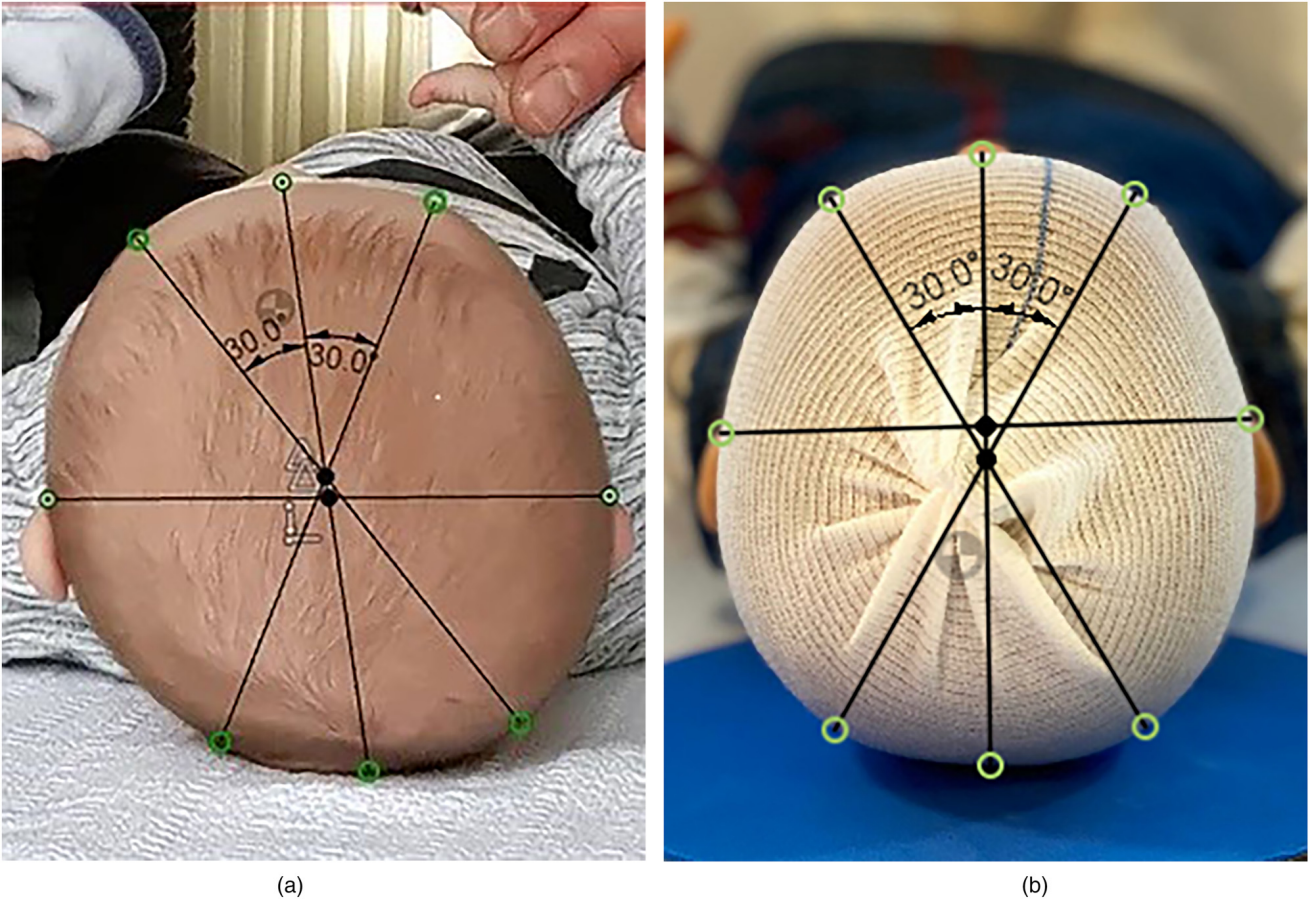
Visualization of the anatomical landmarks. CVAI = (large diagonal − short diagonal)/(short diagonal)×100%. CI = (EarEar)/(NoseOcciput) × 100%: (a) without cap and (b) with cap.

Abbreviations: CVAI, cranial vault asymmetry index; CI, cranial index.

The infant is placed on its back on a table. A photograph of the skull is taken from the vertex view, both the nose and both ears needed to be visible as anatomical landmarks. In case of hair interfering with the examination (eg, visibility of the skull or anatomical landmarks), a standardized cloth skull cap is used. Following this, cranial vault asymmetry index (CVAI) at 30° as a parameter for positional plagiocephaly and cranial index (CI) as a parameter for positional brachycephaly are calculated by an automated algorithm (Figure 1). A horizontal line was drawn touching the anterior helical borders of the ears. The outside of the skull and the midpoint on this line were marked. A vertical line was drawn through the highest point of the nasal dorsum and the midpoint of the horizontal line. The outside of the skull and the midpoint on this line were also marked. From the midpoint of the vertical line 2 oblique lines at 30° were drawn and the outsides of the skull were marked on these lines (Figure 1). A severity scale is connected to SC (Holowka et al., 2017; Di Chiari et al., 2019). CVAI as a parameter for positional plagiocephaly is divided in 5 levels of severity: normal < 3.5; mild ≥ 3.5 and <6.25; moderate ≥ 6.25 and <8.75; severe ≥ 8.75 and <11; very severe ≥ 11. CI as a parameter for positional brachycephaly is divided in 4 levels of severity: normal < 90; mild ≥ 90 and <95; moderate ≥ 95 and <100; severe  ≥ 100.

**Figure 2.**
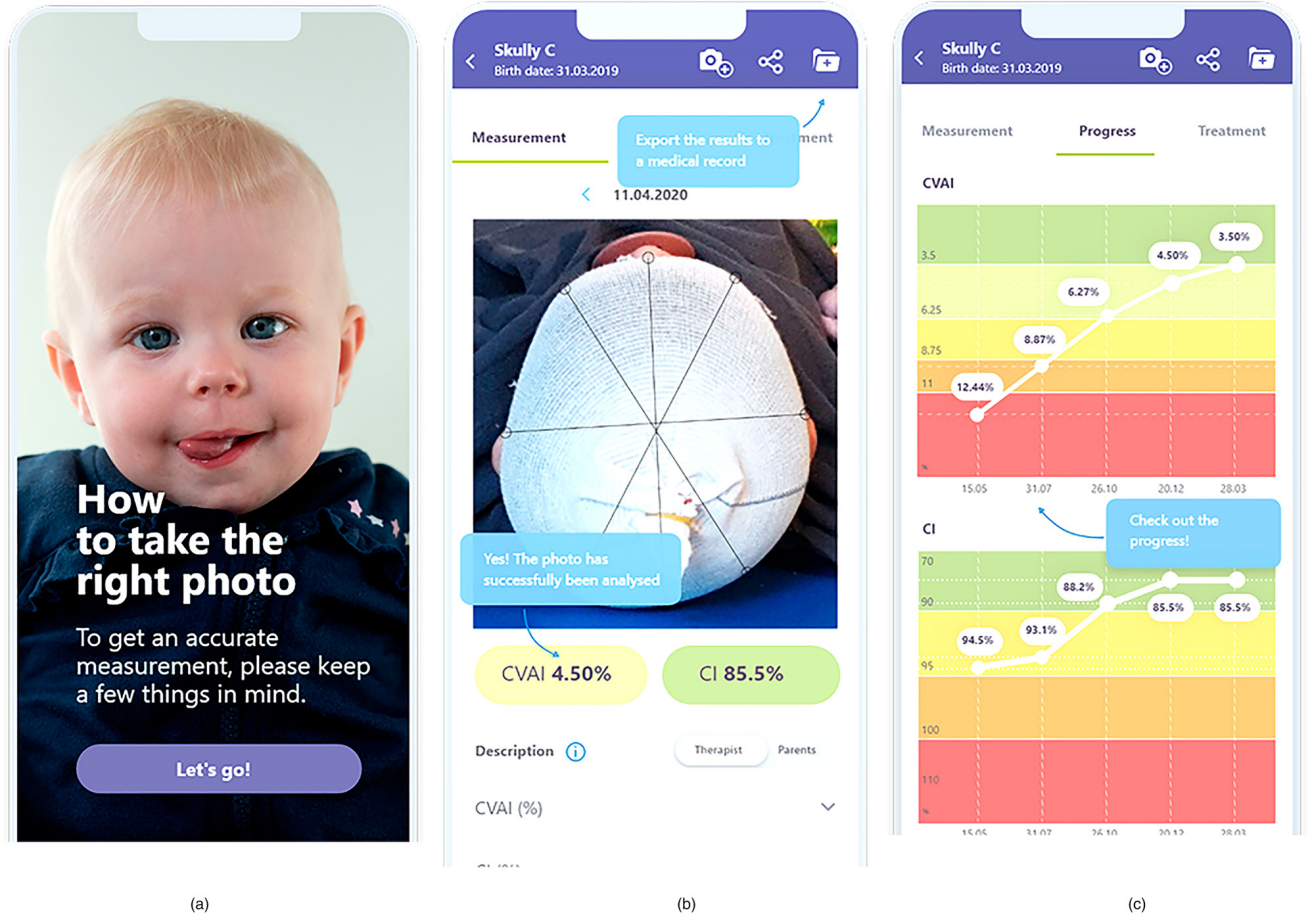
Skully Care (SC) screenshots: start screen “How to take the right photo” (a), presentation of the measurement (b), and clinical follow-up (c).

Results are exported to the medical record (Figure 2).

### Severity Scales

The ODDI, CPI, and CI have 4 levels (eg normal to severe), and the CVAI has 5 levels (eg normal to very severe) (Holowka et al., 2017; Di Chiari et al., 2019). For comparison purposes, the CVAI scale was reduced to 4 levels by equally splitting the middle level and adding the 2 halves to the adjacent levels and renamed to New CVAI. New CVAI as a parameter for positional plagiocephaly is divided in 4 levels of severity: normal < 3.5; mild ≥ 3.5 and <7.5; moderate ≥ 7.5 and <11; severe ≥ 11.The remaining cut-off points are comparable; ODDI (at 40°) 104, 108, and 112 and New CVAI (at 30°) 3.5, 7.5, and 11.

### Duration of Measurement

In 10 cases, duration of measurement of PCM and SC was established. In PCM, the making of the thermoplastic band including anatomical landmarks on the child's head was timed in minutes, as well as the extraction of ODDI and CPI values. In SC, the making of the photograph with the smartphone was timed in minutes, as well as the extraction of CVAI and CI.

### Statistics

Pearson correlation coefficient (2-tailed) was used to determine correlation between CVAI (used in SC) and ODDI (used in PCM) and between CI (used in SC) and CPI (used in PCM). The accepted guidelines for interpreting the correlation coefficients are: +1 indicates a perfect positive linear relationship, −1 indicates a perfect negative linear, and 0 indicates no linear relationship (Ratner, 2019). The size of a correlation coefficient can be interpreted as follows: negligible correlation (0.00-0.30), low (0.30-0.50), moderate (0.50-0.70), high (0.70-0.90), and very high (0.90-1.00) (Hinkle et al., 2003).

The Mann–Whitney *U* test (2-tailed) was used to determine the difference of time consumption between PCM and SC for the “making” phase, for the “analysis” phase, and for the total procedure.

Statistical analyses were performed using the Statistical Package for the Social Sciences (IBM SPSS) for Windows (Version 26, IBM Inc.). Statistical significance was set at a *P* value ≤.05.

The severity scales of both methods are compared and related.

The study was deemed a retrospective clinical study and did not require formal research ethics approval under the Medical Research Involving Human Subjects Act (WMO).

## Results

### Patients

A total of 60 skull shape analyses were performed (14 girls vs 46 boys). Mean age at analysis was 4.6 months (1.1-11.0; SD = 2.3).

### Comparison of SC and PCM

**Figure 3.**
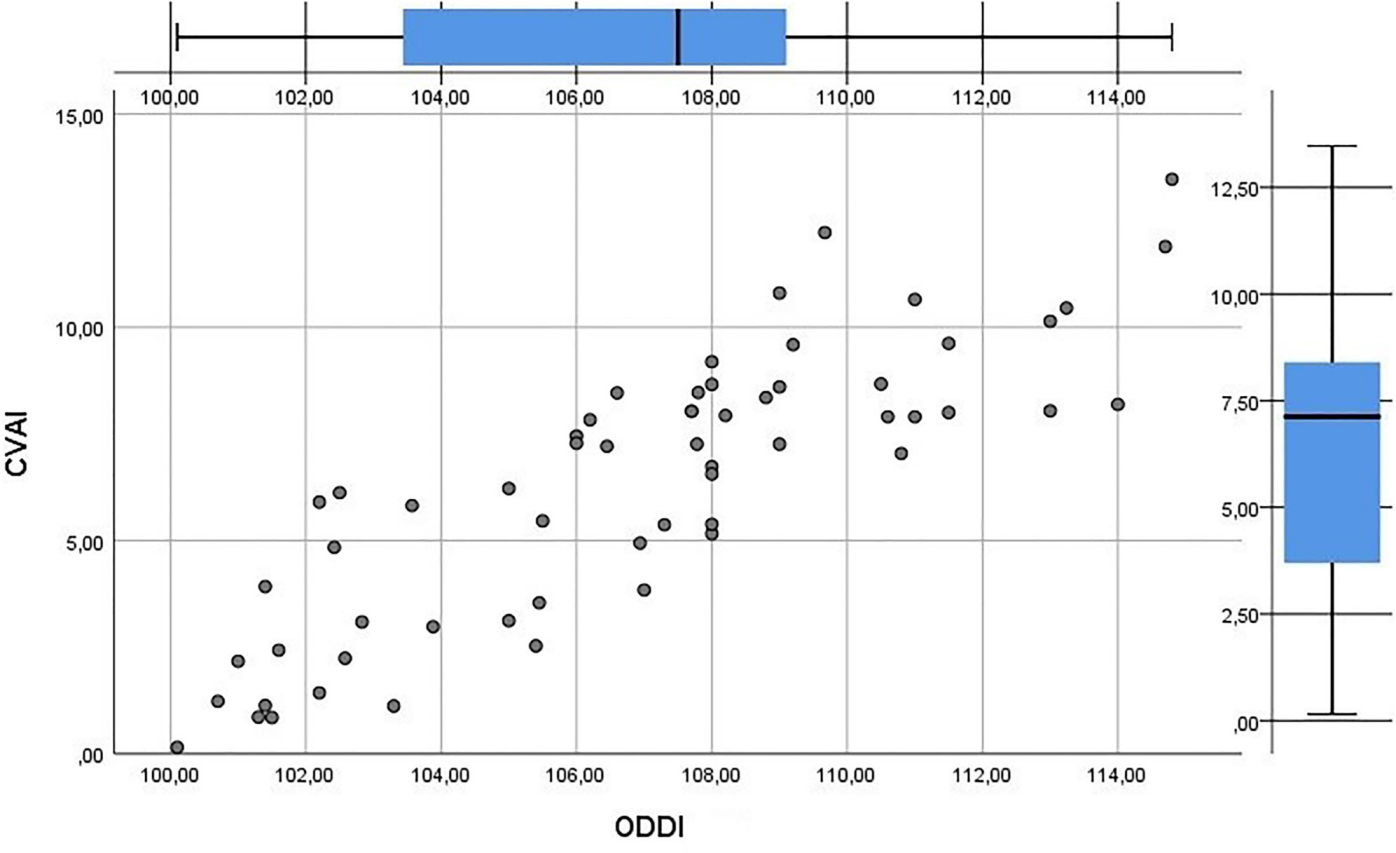
CVAI (used in SC) related to ODDI (used in PCM).

Abbreviations: CVAI, cranial vault asymmetry index; SC, Skully Care; ODDI, oblique diameter difference index; PCM, plagiocephalometry.

Mean CVAI (used in SC) was 6.33 (0.15-13.47; SD = 3.19) and mean ODDI was 106.9 (100.1-114.8; SD = 3.9). High correlation was found between CVAI and ODDI (*r* = 0.849; *P* < .01; Figure 3).

**Figure 4.**
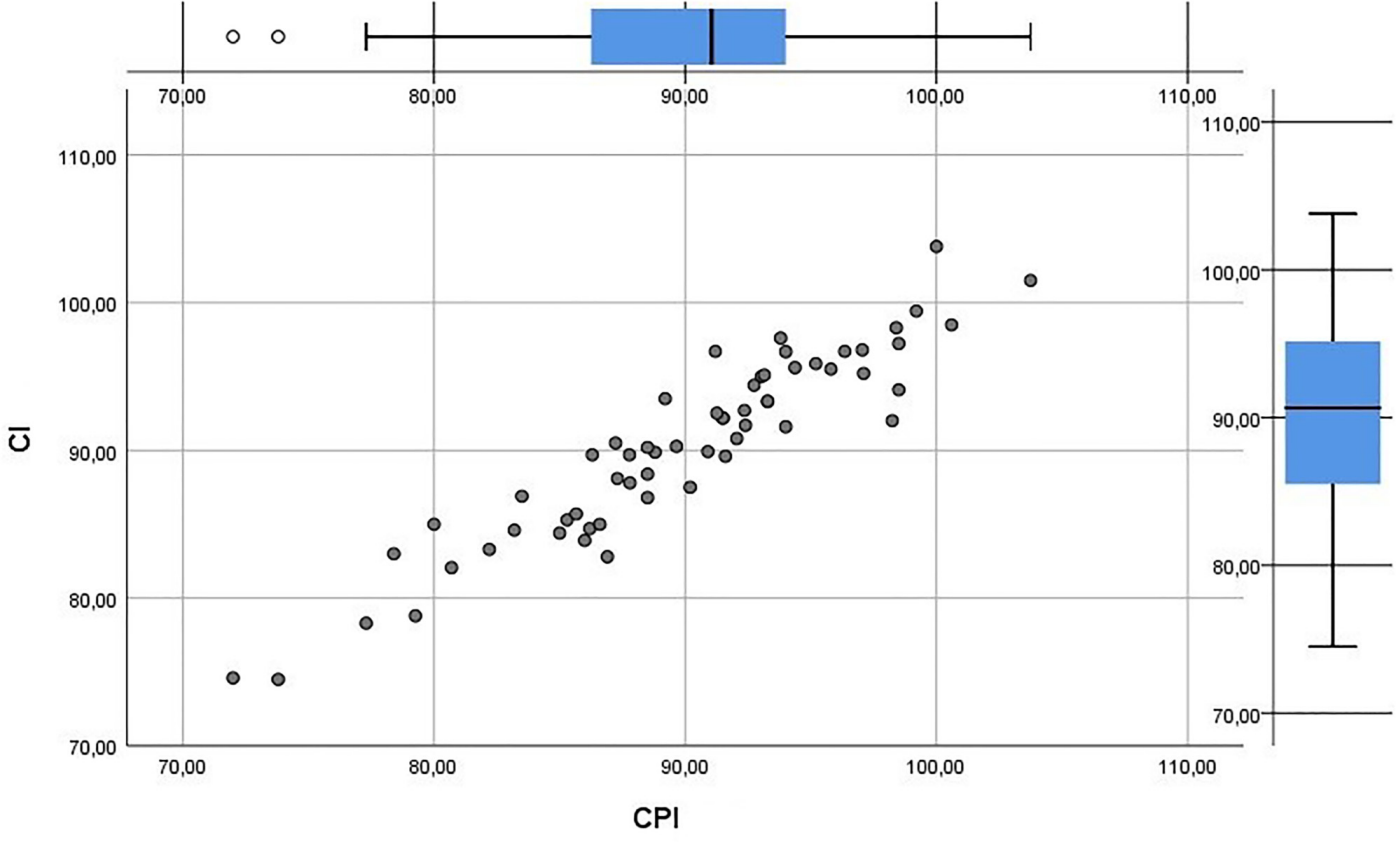
CI (used in SC) related to CPI (used in PCM).

Abbreviations: CI, cranial index; SC, Skully Care; CPI, cranial proportional index; PCM, plagiocephalometry.

Mean CI (used in SC) was 90.4 (74.5-103.8; SD = 6.3) and mean CPI was 89.9 (72.0-103.8; SD = 6.6). Very high correlation was found between CI and CPI (*r* = 0.938; *P *< .01; Figure 4).

The severity scales were compared for positional plagiocephaly (New CVAI and ODDI; Table 1) and for positional brachycephaly (CPI and CI; Table 2). When comparing the New CVAI with ODDI a complete level match is seen in 62% (37 of 60), in 38% 1 level difference (23 of 60), and in 0% (0 of 60) more than 1 level difference. When comparing CI with CPI a complete level match is seen in 73% (44 of 60), in 27% 1 level difference (16 of 60), and in 0% (0 of 60) more than 1 level difference.

**Table 1.** Comparison of the 4 Level New CVAI (Used in SC) and the 4 Level ODDI (Used in PCM).

	ODDI–PCM			
New CVAI–SC	Normal	Mild	Moderate	Severe
Normal	12	2		
Mild	5	10	6	
Moderate		5	13	4
Severe			1	2

*Note.* Dark grey shading is used to highlight a perfect match and light grey shading is used to highlight a 1 level difference.

Abbreviations: CVAI, cranial vault asymmetry index; SC, Skully Care; ODDI, oblique diameter difference index; PCM, plagiocephalometry.

**Table 2.** Comparison of the 4 Level CI (Used in SC) and the 4 Level CPI (Used in PCM).

	CPI–PCM			
CI–SC	Normal	Mild	Moderate	Severe
Normal	24	3		
Mild	4	10	2	
Moderate		6	8	1
Severe				2

*Note. *Dark grey shading is used to highlight a perfect match and light grey shading is used to highlight a 1 level difference.

Abbreviations: CI, cranial index; SC, Skully Care; CPI, cranial proportional index; PCM, plagiocephalometry.

PCM measurement took significantly more time than SC for all phases (*P *< .001). The making phase in PCM took a mean time of 13 min (minimum = 7; maximum = 17; SD = 3.1; *n* = 10) and in SC it took a mean time of 2 min (minimum = 1; maximum = 5; SD = 1.3; *n* = 10) (*P *< .001). The analysis phase in PCM took a mean time of 16 min (minimum = 12; maximum = 20; SD = 16.2; *n* = 10) and in SC it took a mean time of 2 min (minimum = 1; maximum = 6; SD = 1.6; *n* = 10) (*P *< .001). The total measurement in PCM took a mean time of 29 min (minimum = 19; maximum = 37; SD = 5.8; *n* = 10) and in SC it took a mean time of 4 min (minimum = 2; maximum = 8; SD = 1.9; *n* = 10) (*P *< .001).

## Discussion

New innovative technologies such as smartphone applications enable us to improve patient care and make it more efficient and more child convenient.

Primary diagnosis of positional skull deformities is based on clinical history taking and physical examination (Mathijssen, 2015). Due to the need for quantification of severity, PCM was introduced. With the introduction and validation of this method, scientific analysis of outcome was enabled. However, the method needs a direct skin contact with a thermoplastic strip heated to 60°C on the newborn and young child and is laborious and time-consuming (van Vlimmeren et al., 2006; van Adrichem et al., 2008). Additionally, a photographic assessment showed high correlation with anthropometrically derived values, but the method remained laborious and time-consuming, and is therefore not practical for daily clinical practice (Schaaf et al., 2010).

The present study shows a high correlation between the SC application on a smartphone and PCM in quantification of positional plagiocephaly. Additionally, a very high correlation was found between SC and PCM in quantifying positional brachycephaly. Therefore, validation can be marked as successful.

Both SC and PCM have a severity scale for plagiocephaly and brachycephaly; these severity levels are indicative for treatment options by pediatric physiotherapists in the Netherlands. However, in decision-making both SC and PCM can help but are never solely conclusive. Other factors such as positional preference of the head, esthetic appearance, age of the child, and parents’ opinion on treatment are also important. No study proved the validity of these severity scales in clinical decision-making. The power of SC and PCM is recording change over time, for example, during treatment or spontaneously (van Vlimmeren et al., 2008; van Wijk et al., 2014). Very small differences in the exact value might result in a different severity scale, which makes a difference of 1 scale less important. A difference of 2 scales indicates a significant discrepancy. Therefore, the exact values of ODDI, CVAI, CPI, and CI are quantitative and more precise than the severity levels and the most important parameters in establishing validity. CVAI has 5 severity levels, in contrast to ODDI, CPI, and CI that have 4 levels. To make a comparison between ODDI and CVAI, we introduced a 4-level CVAI scale by equally splitting the middle level and adding the 2 halves to the adjacent levels and renamed it New CVAI. By comparing the 2 scales in positional plagiocephaly no difference of more than 1 level was seen; in 62% an exact match was seen. In positional brachycephaly the match was even better with no difference of more than 1 level and an exact match in 73%. No difference of 2 scales, indicating a significant discrepancy, was seen.

SC is easily applicable and can even be performed by the patient's parents (during the Dutch lockdown for coronavirus disease-2019 it turned out to be a good solution for monitoring the patients). This has the advantage of reducing visits to the outpatient clinic, but the infant can still be optimally monitored.

Reducing the time of measurement from 29 min for PCM to 4 min for SC improves efficiency of care. Cost reduction is enhanced by less time effort of the physiotherapist. It is also more efficient for the parents because the therapy plan can be discussed during the first visit to the outpatient clinic, reducing the number of visits to the outpatient clinic by 1. The simplicity of the SC method makes introduction to primary health care by general practitioner or primary child health care centers conceivable.

Both SC and PCM are primarily developed for quantifying positional cranial deformities and not to differentiate between craniosynostosis and positional cranial deformities. Differential diagnosis of craniosynostosis and positional cranial deformities is mainly by medical interview and physical examination (Bredero-Boelhouwer et al., 2009; Feijen et al., 2012; Mathijssen, 2015; Ghizoni et al., 2016). Both methods, however, are used to monitor effect of treatment, for example, physiotherapy. If no or minimal improvement of cranial shape is seen (mostly before 5 months of age), craniosynostosis is suspected and evaluation by a dedicated center is indicated. Recently, the Utrecht Cranial Shape Quantifier proved to differentiate between all types of simple craniosynostosis (Kronig et al., 2020a, 2020b). Future research is planned to implement the used analysis algorithm in SC to use it as a diagnostic tool between craniosynostosis and positional cranial deformations.

A limitation in using photographs for skull analysis might be obvious hair growth. It covers the ears and blurs the skull line. Wearing a standard cloth cap presses the hair on the skull line, enabling a reliable SC measurement.

A second limitation might be the slightly different angle of plagiocephaly analysis in CVAI and ODDI. CVAI uses a 30° angle on the anterior–posterior line and ODDI a 40° angle. Many studies use CVAI, PCM uses ODDI. However, this study shows a high correlation between the CVAI-based SC and the ODDI-based PCM.

A third limitation might be that applications on smartphones can change over time. We advise to repeat the validity study if the algorithm of the SC application shows substantial changes. Furthermore, a stable internet connection is needed.

We can conclude that the SC application on a smartphone was validated and correlated to PCM, which is the gold standard in daily physiotherapy practice. High and very high correlations between SC and PCM were found. The combination of not only simplicity and validity, but also speed and user and child convenience makes SC a promising craniofacial measuring method in daily practice. SC has the potential to be the modern successor for analyzing positional cranial deformities.
